# Lgr6+ stem cells and their progeny in mouse epidermis under regimens of exogenous skin carcinogenesis, and their absence in ensuing skin tumors

**DOI:** 10.18632/oncotarget.13436

**Published:** 2016-11-17

**Authors:** Gerline C. van de Glind, Heggert G. Rebel, Jacoba J. Out-Luiting, Wim Zoutman, Cornelis P. Tensen, Frank R. de Gruijl

**Affiliations:** ^1^ Department of Dermatology, LUMC, Leiden, The Netherlands

**Keywords:** stem cells, Lgr6, lineage tracing, UV, skin carcinogenesis

## Abstract

Lgr6+ cells have been identified as a novel class of proliferating (Ki67+) stem cells in mouse epidermis. We investigated their response to UV exposure in *Lgr6-EGFP-Ires-CreERT2/R26R-LacZ* haired and hairless mice and whether they become initiating cells of UV- or chemically induced skin tumors. UV overexposure erased Lgr6+ cells (EGFP+) from the interfollicular epidermis (IFE), but - as after wounding - they apparently repopulated the IFE from the hair follicles. Under sub-sunburn chronic UV exposure, Lgr6+ cells and their progeny (LacZ+ after pulse of tamoxifen) diminished strongly in the IFE. Although the inter-tumoral IFE clearly showed Lgr6 progeny, none of the UV- or chemically induced tumors (n = 22 and 41, respectively) appeared to be clonal expansions of Lgr6+ stem cells; i.e. no Lgr6+ cells or progeny in the proliferating tumor bulk. In checking for promoter methylation we found it to occur stochastically for the *EGFP-Cre* cassette. *Lgr6* mRNA measured by qPCR was found to be diminished in skin tumors (also in UV tumors from wt type mice). The ratio of *Lgr6/Ki67* was significantly reduced, pointing at a loss of Lgr6+ cells from the proliferative pool. Our data show that Lgr6+ cells are not major tumor-initiating cells in skin carcinogenesis.

## INTRODUCTION

Skin cancer is the most common cancer in white-skinned European ancestral populations; the incidence of non-melanoma (squamous cell carcinoma and basal cell carcinoma) and melanoma skin cancer is rising [1, 2]. UV exposure plays an important role in the etiology of these skin cancers [3, 4]. Cancer is a multistep process (multiple ‘hits’ or mutations) that eventually results in a malignant cell sprouting into a cancerous growth [5]. Since stem cells are long residing cells, these cells are prime candidates to accumulate oncogenic changes over time. One of the more recently identified stem cell populations in the skin is that of Lgr6+ cells [6]. We therefore posed the question whether these stem cells are targeted in experimental skin carcinogenesis by exogenic agents.

Lgr6+ stem cells are located in the isthmus region of the hair follicle (HF), in the sebaceous gland (SG) and in the interfollicular epidermis (IFE) [6–9]. Snippert et al identified these stem cells in the skin and found that they are a unique population not expressing CD34 or K15 [6]. The Lgr6+ cells in the IFE, isthmus and SG are able to maintain their respective compartments in the long term [9]. It was recently shown that Lgr6+ cells increase in numbers in the IFE of mice from birth to adulthood (8 weeks of age) [9]. Like its family members Lgr4 and 5, the Lgr6 receptor (leucine-rich repeat-containing G protein-coupled receptor 6) contains a seven transmembrane region and an ectodomain with 13 leucine-rich repeats [10], and after binding R-spondin it can enhance canonical Wnt/β-catenin signaling [11].

We have previously demonstrated that experimental, UV-induced squamous cell carcinomas (SCCs) originate from the IFE [12]. A recent study of ours [13] implicated quiescent stem cells in UV-induced tumor initiation. These cells accumulate and retain DNA damage (cyclobutane pyrimidine dimers) during low level chronic UV exposure, which increases the risk of mutagenesis when these cells are forced to divide. Additionally, the continuously actively dividing Lgr6+ stem cells in the IFE are likely UV-targeted candidate cells from which SCCs may originate. To investigate this latter possibility we carried out experiments on how these Lgr6+ stem cells and their progeny respond to UV radiation and whether they drive UV carcinogenesis. Next to UV carcinogenesis, the Lgr6+ stem cells may be targeted in chemical skin carcinogenesis, which we studied in additional experiments.

The established model for UV carcinogenesis is chronic UV exposure of SKH1 hairless mice [14]. After daily genotoxic challenges from sub-sunburn UV dosages these mice develop SCCs similar to those in humans. These tumors in mice harbor UV-signature mutations in *p53* similar to those in SCCs in humans [15]. The widely used model for chemical skin carcinogenesis is the two-stage model: a single application of a genotoxic agent (e.g. 7,12-Dimethylbenz[a]anthracene, DMBA) initiates tumors and subsequent repeated applications of a (non-genotoxic) irritant (most commonly 12-O-Tetradecanoylphorbol-13- acetate, TPA) promotes further tumor development (outgrowth) [16]. The tumors that develop are mainly papillomas with *H-Ras* mutations [17, 18] and to a much lesser extent SCCs. We have used both models to investigate the role of Lgr6+ stem cells and their progeny in skin carcinogenesis.

In the present study we used hairless and shaven haired heterozygous transgenic *Lgr6-EGFP-Ires-CreERT2/R26R-LacZ* mice containing a *Rosa26-LacZ* reporter for lineage tracing. Lgr6-expressing cells were EGFP+ and, after administering tamoxifen, the progeny could be detected as LacZ+ cells (i.e. with β-galactosidase activity which cleaves X-gal leaving a blue product). These transgenic mice were subjected to genotoxic UV regimens that are physiologically relevant to humans. One regimen was daily sub-acute exposure for 4-8 weeks inducing epidermal hyperplasia, and the other regimen was a single tolerable UV overexposure that largely ablated the epidermal basal layer by apoptosis but left the overlying layers intact (i.e. no wounding). In the UV carcinogenesis experiments the hyperplasia-inducing UV regimen was prolonged to develop tumors in hairless mice (see Materials and Methods). Haired and hairless mice subjected to chemical carcinogenesis received a single initial DMBA application followed by TPA applications twice a week. We studied skin samples (cross sections, whole mounts and epidermal sheets) and tumor samples to investigate the response of Lgr6+ stem cells and their progeny (“Lgr6 progeny” for short) to the ablative and carcinogenic regimens (see time lines for the different experiments in Supplementary Figure S1).

## RESULTS

### Lgr6+ stem cells are present in the skin of hairless mice

**Figure 1:**
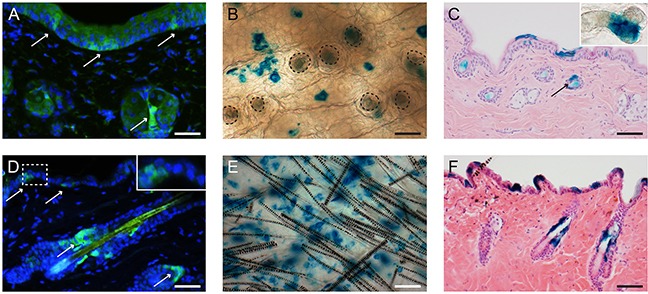
In homeostasis Lgr6+ stem cells and their progeny are present in the lower part of the HF remnants and in the IFE of hairless mice A-C. and in the isthmus and IFE of haired mice D-F Skin sections of *Lgr6-EGFP-Ires-CreERT2/R26R-LacZ* mice were stained for EGFP to detect Lgr6+ stem cells (A+D, arrows). Skin whole mounts (B+E; HF orifices contoured in B), cross sections (C+F) and epidermal sheets (insert HF remnant in C) were stained (blue) for LacZ expression (8-9 weeks after tamoxifen) to detect Lgr6 progeny. Scale bar in E = 100 μm, scale bar in B, C, F= 75 μm, scale bar in A+D= 50 μm

We first ascertained whether Lgr6+ stem cells were present in the epidermis of (transgenic) hairless mice (see Figure 1, also for comparison with haired mice). To this end, haired *Lgr6-EGFP-Ires-CreERT2/R26R-LacZ* mice were backcrossed into a hairless background using albino SKH-1 mice. The progeny was viable and did not show a specific phenotype. As in SKH-1 mice, HFs in hairless *Lgr6-EGFP-Ires-CreERT2/R26R-LacZ* mice appeared to be arrested in catagen. HF remnants were connected to deep-seated cysts in the dermis (presumed bulb remnants) [12]. Lgr6-expressing stem cells (EGFP+ in Figure 1A) were present in the IFE and near the bottom of HF remnants (region of sebaceous glands). Lgr6 progeny (LacZ+) was found in the lower part of the HF remnant (see Figure 1C). We also found Lgr6 stem cell progeny (LacZ+) in the IFE (see Figure 1B + 1C).

### Lgr6+ stem cells and their progeny repopulate the interfollicular epidermis after UV overexposure

**Figure 2:**
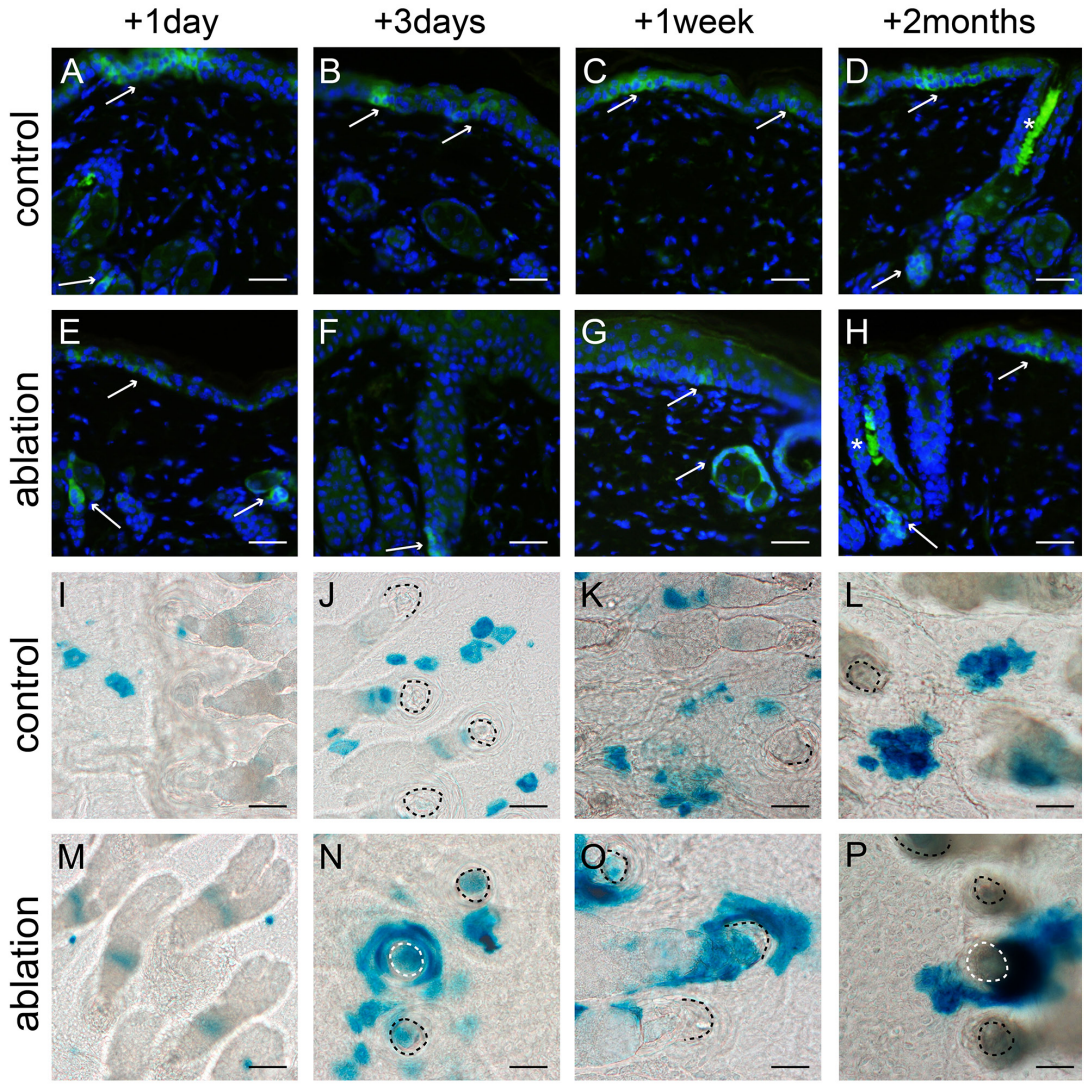
Lgr6+ stem cells and their progeny in hairless mice after UV overexposure Anti-EGFP staining was used to localize the Lgr6+ stem cells **A-H**. and epidermal sheets were stained for LacZ+ progeny **(I-P)** at different time points (representative pictures are shown). The control mice (A-D) showed clear EGFP+ cells in the IFE and at the bottom of the HF remnants (A+D see arrows). The LacZ+ Lgr6 progeny spread in the IFE in control skin I-L. One day after UV overexposure we still observed some EGFP-expression in the IFE (E), this expression was lost after 3 days (F) and reappeared sparsely after 1 week (G). EGFP expression at the bottom of the HF remnants was present at all time points (E-H). After UV overexposure Lgr6 progeny was more prevalent in HF remnants and in the IFE progeny was more associated with the hair follicle remnants M-P. compared to the controls. Scale bars 50 μm. *= auto fluorescence/ false positivity caused by keratins.

**Figure 3:**
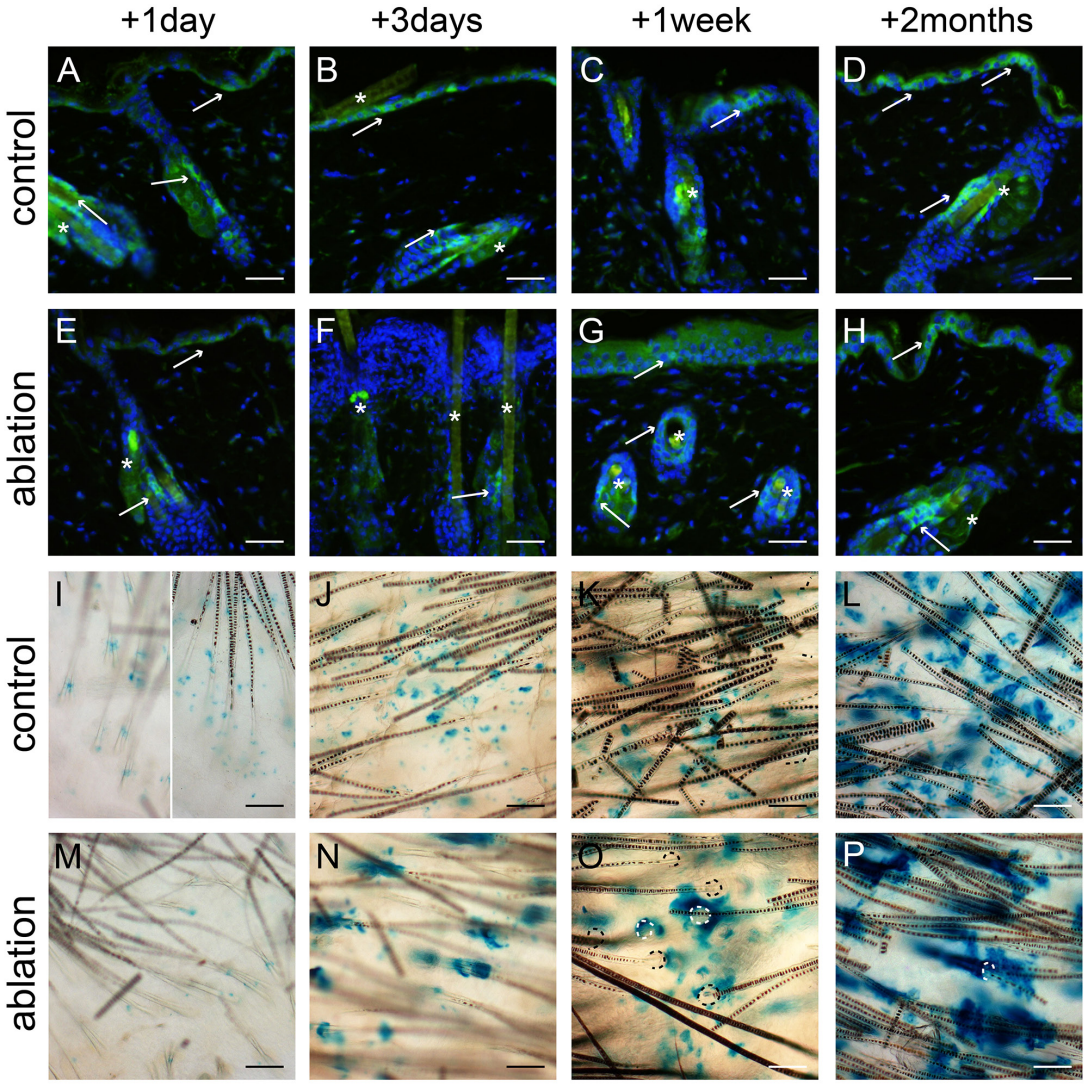
Lgr6+ stem cells and their progeny in haired mice after UV overexposure Anti-EGFP staining was used to localize the Lgr6+ stem cells **A-H**. and whole mounts were stained for LacZ+ progeny **I-P**. at different time points. We observed some autofluorescence in hairs. The control mice (A-D) showed clear EGFP+ cells in the IFE and in the isthmus (A-D, see arrows). Lgr6 progeny built up in the HFs and islands in the IFE (I-L). One day after UV overexposure we still found some EGFP expression in the IFE (E, in apoptotic basal layer), that expression was lost after 3 days (F, with inflammatory infiltrate in papillary dermis) and reappeared sparsely after 1 week (G). We found EGFP expression in the isthmus at all time points (E-H). One day after UV overexposure we only observed some LacZ+ cells in the HFs and not at all in the IFE (I). From three days after UV overexposure the Lgr6 progeny was found in the IFE, more associated with HFs remnants (N-P) compared to the controls. HF orifices indicated by dotted lines (O+P). Scale bars = 50 μm. *= background autofluorescence in sebaceous glands and from hairs.

We ablated a large part of the epidermal basal layer using a tolerable UV overexposure (3.2 and 2.5 kJ/m^2^ UV for haired and hairless mice, respectively; see Material and Methods). This dose induced massive apoptosis in basal cells but left the overlying cell layers intact [12]. We stained for Lgr6+ stem cells, using an anti-EGFP antibody, in skin samples taken at different time points after UV overexposure; see Figures 2 and 3(E-H) for hairless and haired mice, respectively. EGFP-expression was much less prevalent but still weakly present in the apoptotic basal layer of the IFE 1 day after UV overexposure both in haired and hairless mice. However, 3 days after UV overexposure the IFE no longer showed any EGFP+ expression. From 1 week after UV overexposure onward EGFP-expressing Lgr6+ cells reoccurred in the IFE. In the control samples that did not receive a UV overexposure we found EGFP+ cells in the IFE equally at all time points (Figures 2 and 3A-D). The EGFP+ cells in the isthmus of haired mice and in HF remnants of hairless mice were present at all time points and did not appear to be influenced by the overexposure. Also, Lgr6 progeny (LacZ+) remained present in HFs (isthmus) of haired and HF remnants of hairless mice. A lack of progeny in the IFE appeared only in haired mice 1 day after exposure (Figure 3M). In haired mice Lgr6 progeny reappeared in the IFE 3 days after UV overexposure (Figure 3N) whereas in hairless mice it never fully disappeared, already reappearing early after 1 day (Figure 2M). From day 3 onward progeny was more associated with HFs in the overexposed groups than in controls (Figures 2 and 3). This clearly indicated outgrowth of the progeny from the HFs to replace the ablated epidermal basal cells. Furthermore, we observed more LacZ+ progeny in the HFs and HF remnants at 3 days and 1 week after UV overexposure all the way up to the rims where HFs are connected to the IFE (Figures 2 and 3N+O). This was indicative of increased proliferation in HFs after overexposure. Eight weeks after the ablative dose, the skin was still perturbed with 2.0 (SEM 0.3) LacZ+ clusters of Lgr6 progeny per mm^2^ in the IFE versus 3.9 (SEM 0.6) in controls of which 52% (SEM 11%) versus 2.5% (SEM 0.4%) were associated with HFs.

### Interfollicular Lgr6 expression is reduced after chronic UV exposure

**Figure 4:**
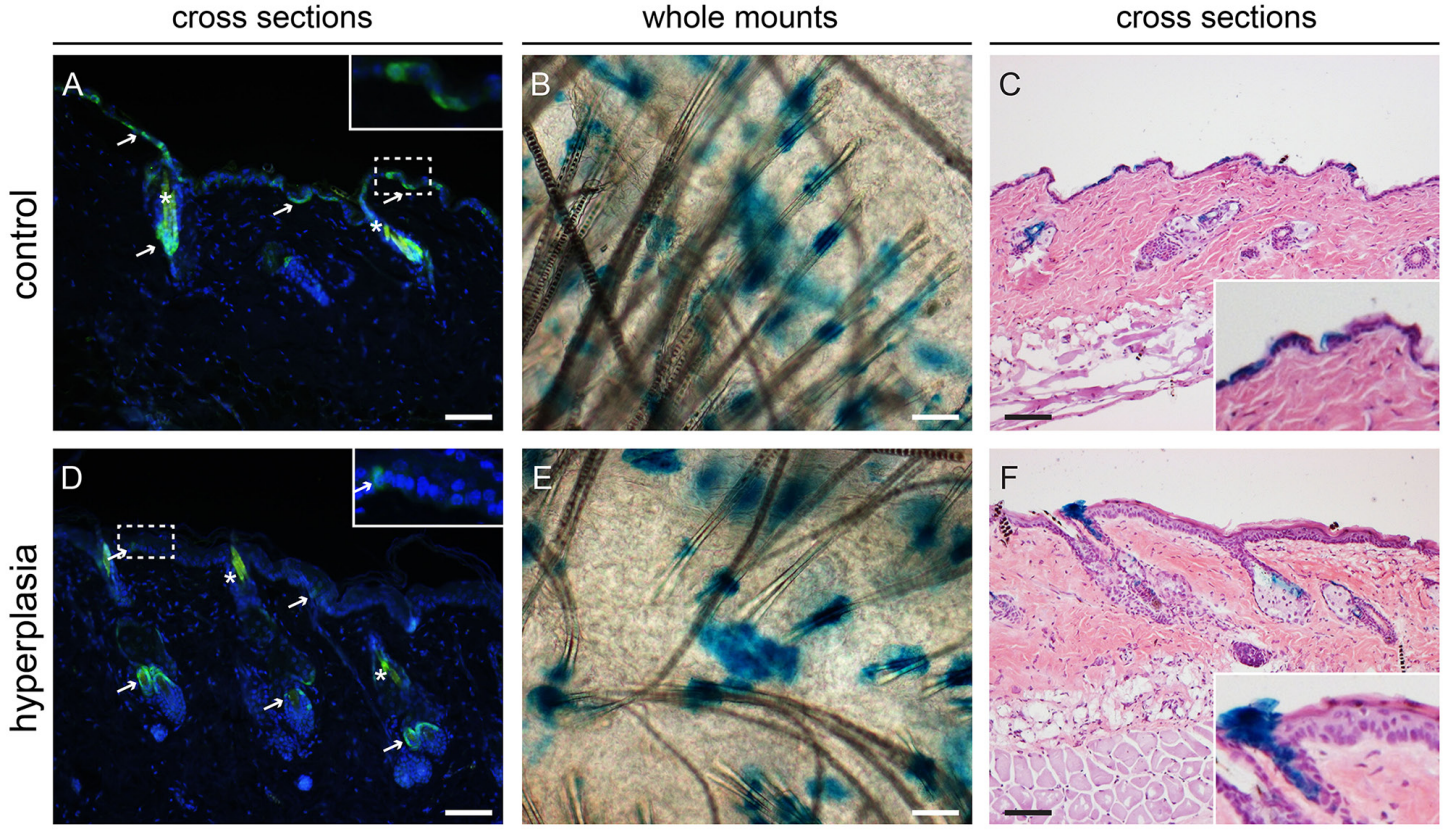
Lgr6+ stem cells and their progeny in haired mice after chronic UV exposure Samples were isolated at the 8-week time point. Anti-EGFP staining was used to localize the Lgr6+ stem cells (in cross sections, **A+D**) and LacZ+ cells as progeny (stained blue in whole mounts in top view, **B+E**. and in cross sections **C+F**). We observed some auto-fluorescence in hairs (*). In control samples we observed patches of EGFP+ cells in the IFE (A), in hyperplastic skin only a few EGFP+ cells were found in the IFE (D). Inserts in A and B are magnifications of dashed frames of the IFE. Lgr6 progeny was found in the isthmus and in islands in the IFE in both control and hyperplastic skin (B,C,E,F). Scale bars = 100 μm.

**Figure 5:**
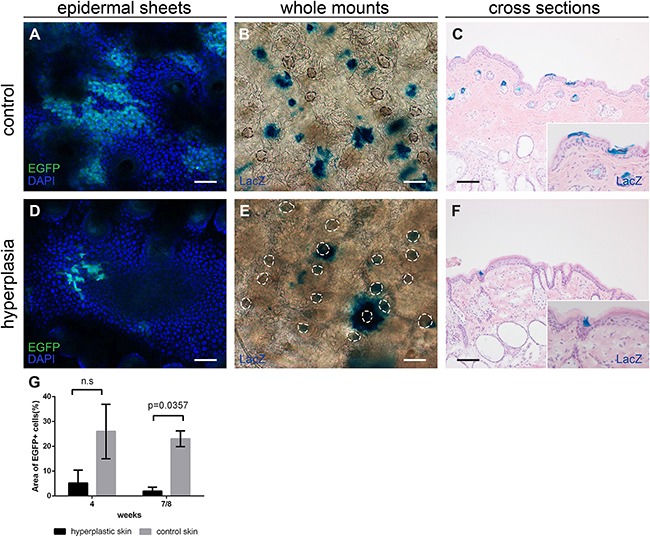
Lgr6+ stem cells and their progeny in hairless mice after chronic UV exposure, samples after 8 weeks of exposure CLSM on epidermal sheets was used to investigate EGFP expression **A+D**. Nuclei were stained with DAPI. In control mice clear EGFP+ islands were visible in the IFE (A), in the UV-hyperplastic skin the cluster of EGFP+ cells were smaller and less frequent (D). The area occupied by EGFP+ cells as a percentage of the area of DAPI+ cells was calculated for 4 and 7-8 weeks, graphs represent mean and SEM **(G)**. Whole mounts **B+E**. and cross sections **C+F**. were stained for LacZ+ progeny. Hyperplastic skin (E+F) showed fewer LacZ+ cell clusters in the IFE and these tended to be larger and more often found associated with HF remnants. Scale bars = 100 μm.

Mice were subjected to daily sub-acute exposure (1 MED/d) for 4-8 weeks to induce epidermal hyperplasia. These mice were sacrificed at the different time points together with their respective controls that did not receive any UV treatment. Upon UV induction of epidermal hyperplasia Lgr6+ stem cells remained in the isthmus of haired mice and in the HF remnants of hairless mice (see Figure 4, hairless data not shown). However, their number was reduced in the IFE (see Figure 4 for haired and Figure 5 for hairless mice). The difference was most strikingly observable in epidermal sheets from hairless mice. In the control samples there were clear large ‘islands’ of Lgr6+ cells (Figure 5A), whereas we only saw some isolated single Lgr6+ cells or small clusters of Lgr6+ cells in the sheets of UV-hyperplastic epidermis (Figure 5D). We measured the EGFP+ area (by number of pixels) within the area populated by epidermal cells (high density of DAPI+ nuclei), and found a significant difference in EGFP+ fractions by area between the control and hyperplastic skin at week 7-8 (p= 0.036, Figure 5G). The reduction of EGFP expression was not a UV-bleaching effect, since this reduction persisted after 6 days without UV exposure. Corresponding observations were made for the progeny of Lgr6+ stem cells. In UV-exposed hairless mice LacZ+ cell clusters also diminished strongly in the IFE: after 8 weeks from 8.6 (SEM 2.7) LacZ+ clusters per mm^2^ in unexposed controls down to 0.09 (SEM 0.08) in the UV-exposed animals. Most of the clusters in the IFE of controls were not associated with HFs, only 0.10 (SEM 0.05) clusters/mm^2^ were. Virtually all clusters not connected to HFs had disappeared from the UV-irradiated IFE (< 0.01/mm^2^), leaving only a low and highly variable number of clusters evidently sprouting from HF remnants (Figure 5B vs 5E). The LacZ+ clusters associated with HF remnants in the hyperplastic IFE tended to be larger than the clusters in unexposed controls. A reduction in LacZ+ clusters was also observed in (shaven) haired mice (Figure 4B vs 4E note that many LacZ+ HFs are present next to LacZ+ cell clusters in the IFE; discrimination under the microscope was actually made easier by varying focus depth between superficial IFE and deeper HFs).

### Interfollicular inter-tumoral Lgr6 progeny

**Figure 6:**
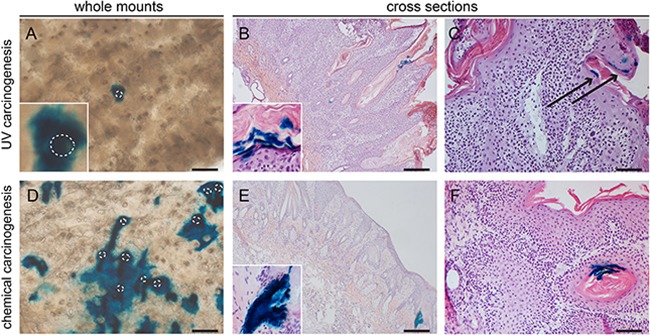
Lgr6 progeny in inter-tumoral skin and skin tumors of hairless mice Samples were stained for LacZ+ cells (UV- carcinogenesis **A-C**; chemical carcinogenesis **D-F**). Whole mount inter-tumoral skin after UV-carcinogenesis (A) showed less Lgr6 progeny in the IFE compared to inter-tumoral skin after chemical carcinogenesis (D). UV-induced tumors showed very sparse labelling in differentiated cells either when lineage tracing was initiated at the beginning of the experiment (B) or when tumors were formed (C); the same holds true for chemically induced tumors (E, F). Some hair follicle-like structures bordering the tumor masses stained LacZ+, with early lineage tracing (E and insert); tumor with late tracing in F. Scale bars = 100 μm (C+F) and 200 μm (A,B,D,E).

**Figure 7:**
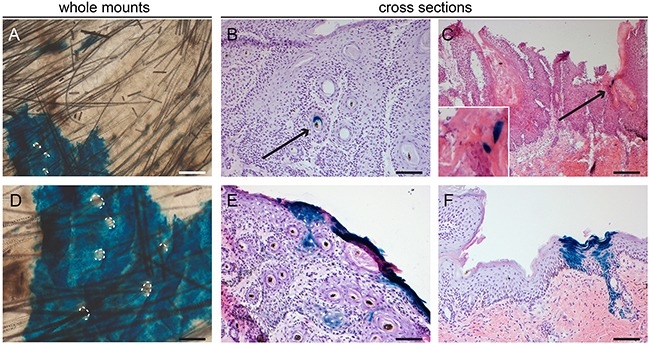
Lgr6 progeny in inter-tumoral skin and skin tumors of haired mice Samples subjected to chemical carcinogenesis were stained for LacZ+ cells. Whole mount inter-tumoral skin after chemical carcinogenesis **A+D**. showed large islands of Lgr6 progeny in the IFE when lineage tracing was induced at the start of the experiment. Hair follicle orifices contoured in A+D of hair follicles that did not stain. Only after early lineage tracing some tumors showed incidental staining in engulfed hair follicle-like structures **B**. We found more abundant staining in uninvolved skin adjacent to the tumors, notice features of proliferating units **F**. When lineage tracing was induced when tumors were formed we found some incidental staining in differentiated cells (**C** and insert). **E**. shows the only tumor (out of 43) in haired mice subjected to chemical carcinogenesis that showed some staining at the outer rim of the tumor with early tracing. Scale bars = 100 μm (B-F) and 200 μm (A).

In the haired and hairless mice from the carcinogenesis experiments we investigated the uninvolved hyperplastic skin adjacent to the tumors. Lgr6 progeny remained present in the HFs and HF remnants. When lineage tracing was started from the beginning of the experiment (i.e. tamoxifen before carcinogenic regimen) we found clear islands of LacZ+ Lgr6 progeny in the IFE (Figures 6A+6D and Figures 7A, 7D, 7F). However, the hairless IFE subjected to UV carcinogenesis (1MED/day >6 months) showed much less progeny (Figure 6A) than that subjected to chemical carcinogenesis (Figure 6D), more similar to the earlier UV-induced hyperplasia (Figure 5E). In stark contrast to UV carcinogenesis, chemical carcinogenesis resulted in large and much more frequent clusters of Lgr6 progeny in the IFE. Virtually all of this progeny was contiguous with that in HFs (all LacZ+) while growing around other LacZ- HFs (Figures 7A, 7D).

### Lgr6+ stem cells are not tumor-driving cells

The main interest of our study was the role of Lgr6+ stem cells and their progeny in the formation of skin tumors. Hence, we subjected *Lgr6-EGFP-Ires-CreERT2/R26R-LacZ* hairless mice to UV-induced skin carcinogenesis and *Lgr6-EGFP-Ires-CreERT2/R26R-LacZ* haired and hairless mice to DMBA and TPA-induced (chemical) carcinogenesis (see also Materials and Methods). In each experiment one group received tamoxifen injections to initiate lineage tracing just prior to starting the carcinogenic regimen and the other group received the injections when tumors (> 4 mm) had formed. Giving the injections at the beginning of the experiments would give us the opportunity to study clonal expansion of a lineage of Lgr6+ stem cells into tumors. In this case the Lgr6 progeny would make up the complete tumor mass, staining completely blue from X-gal cleaved by β-galactosidase, coded for by the *LacZ* gene. Injecting in mice with already established tumors and subsequent tracing for 2-3 weeks would enable determining whether Lgr6+ stem cells in the tumor were driving the growth.

Mice that received the UV-carcinogenic regimen mainly developed (endophytically growing) SCCs and precursors (actinic keratoses). These differed from chemically induced tumors which were mainly (exophytically growing) papillomas. We only found some sporadic Lgr6+ cells (EGFP+) in the bulk of 1 tumor (out of 6 tumors from 4 mice) from the UV carcinogenesis group (data not shown). The tumors obtained with the chemical carcinogenic regimen (n=12 tumors from 7 mice) were all negative. Overall, Lgr6 progeny was present very sparsely and most tumors showed no staining at all (60-70% of chemically- induced tumors and early traced UV tumors, and virtually all late traced UV tumors). In the following paragraphs we give a more detailed description.

In the hairless mice subjected to UV carcinogenesis and traced from the start of the experiment, we observed some signs of progeny in keratin ‘pearls’ and sparse clusters of a few cells in the outer most differentiated layers of the tumors (9 out of 22 tumors from 4 mice, see Figure 6B). Furthermore, some progeny was found in undifferentiated cells in hair follicle-like structures at the edge of the tumors (data not shown). However, the proliferative bulk of the tumors was negative. When we started the lineage tracing when tumors had already formed, only 1 tumor showed LacZ+ cells, and these cells were again very sparse and terminally differentiated (Figure 6C). All other tumors were negative (n=43 from 7 mice).

Haired and hairless mice subjected to chemical carcinogenesis showed similar results. With early initiation of lineage tracing Lgr6 progeny appeared in sparse clusters of a few cells in the outermost differentiated cell layers. Remnants of progeny (blue X-gal staining) were observed in some keratin ‘pearls’ or hair follicle-like structures in the tumors (most likely inclusions of normal cells). This occurred in 6 out of 20 tumors from 3 haired, and in 6 out of 21 tumors from 5 hairless mice (Figure 6E and Figure 7). And again, Lgr6 progeny was present in HFs bordering the tumors. In one tumor from a haired mouse subjected to chemical carcinogenesis and traced from the start of the experiment, we found more abundant Lgr6 progeny at the outer rim of the tumor but not in the tumor bulk (see Figure 7E). In contrast to UV-induced tumors, chemically induced tumors showed some Lgr6 progeny in sparse clusters of a few differentiated cells up on starting the tracing when the tumors had formed (haired 8 out of 23, hairless 6 out of 19, from 6 and 4 mice respectively; see Figure 6F and Figure 7C).

### The Lgr6 promoter becomes methylated in Lgr6-EGFP-Ires-CreERT2/R26R-LacZ mice but not in wild type mice

Since Lgr6+ stem cells and their progeny were absent in the tumor bulk, we posed the question whether EGFP and LacZ expression could have been silenced by aberrant methylation of the promoters involved. Therefore, we checked the methylation status of these promoters in several skin samples and tumors. After identifying CpG islands, promoter hypermethylation was evaluated for *Lgr6* and *Rosa* (*R26R*) in samples from wild type (wt) SKH-1 and C57Bl/6 mice, *Lgr5-EGFP-Ires-CreERT2/R26R-LacZ* and *Lgr6-EGFP-Ires-CreERT2/R26R-LacZ* mice (see Table 1 and Supplementary Table SII). Three of the aforementioned strains do not contain the *EGFP-Ires-CreERT2* cassette in the *Lgr6* locus and therefore carry only wt *Lgr6* loci. Independent of treatment, aberrant promoter hypermethylation was only observed in part of the *Lgr6-EGFP-Ires-CreERT2/R26R-LacZ* samples (see Supplementary Figure S2). These included untreated skin samples (3 methylated out of 8, see Table 1 and Supplementary Figure S2). Combined with the unmethylated status of all wt *Lgr6* samples, these results indicate that *de novo* methylation was exclusively evoked by the insertion of the *EGFP-Ires-CreERT2* cassette. This points to a possible monoallelic silencing of the cassette by methylation of its *Lgr6* promoter (we never observed complete promoter hypermethylation indicating that the wt *Lgr6* promoter remained unmethylated, see Supplementary Figure S2). No differences were observed between haired and hairless *Lgr6-EGFP-Ires-CreERT2/R26R-LacZ* mice nor between chemical and UV carcinogenesis (hence the condensed form of Table 1, a more stratified version in Supplementary Table SII). All chemically induced tumors and most of UV-induced tumors from *Lgr6-EGFP-Ires-CreERT2/R26R-LacZ* mice showed monoallelic methylation. Aberrant hypermethylation of the *R26R* promoter was not observed in any of the samples (data not shown).

**Table 1:** Samples tested for methylation of the Lgr6 promoter

sample group	# samples tested	# with part ofLgr6 promotersmethylated	% samples showingmethylation
Skin untreated (mice with wt Lgr6 locus)	10	0	0
skin untreated (*Lgr6-EGFP-Ires-CreERT2/R26R-LacZ mice*)	16	7	44
skin treated with UV 30 days 1 MED (mice with wt Lgr6 locus)	4	0	0
skin treated with UV 4-8 weeks 1 MED (*Lgr6-EGFP-Ires-CreERT2/R26R-LacZ mice*)	14	6	43
inter-tumoral skin (*Lgr6-EGFP-Ires-CreERT2/R26R-LacZ* mice)	16	14	88
Tumors (*Lgr6-EGFP-Ires-CreERT2/R26R-LacZ mice*)	27	26	96
Tumors (mice with wt Lgr6 locus)	12	0	0

Aberrant methylation was detected by MS-MCA. We tested samples from mice with a wt *Lgr6* locus and samples from haired and hairless *Lgr6-EGFP-Ires-CreERT2/R26R-LacZ* mice. Samples of these last two groups were lumped together since no differences were observed; the same was true for tumors from the chemical and UV carcinogenesis experiments. None of the samples from the *Lgr6-EGFP-Ires-CreERT2/R26R-LacZ* mice showed complete methylation of *Lgr6* promoters, next to a peak from methylated promoters there was always a peak from unmethylated promoters.

### Lgr6 expression is reduced in skin tumors

**Figure 8:**
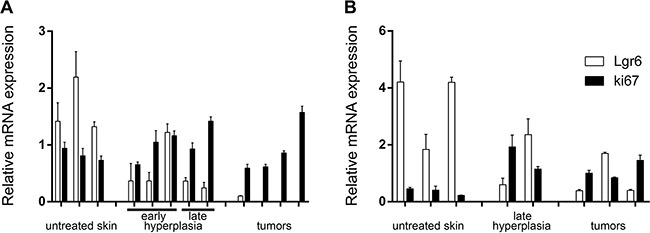
Relative mRNA expression of Lgr6 in hairless A. and haired B. mice Gene expression of *Lgr6* and *Ki67* was measured by qPCR and normalized against stably expressed reference genes (see Material and Methods). RNA was isolated from hairless samples taken from untreated skin, hyperplastic skin induced by UV and UV-induced tumors (A). *Lgr6* gene expression was significantly reduced in UV-induced tumors compared to the untreated and hyperplastic skin samples (p=0.004). RNA was also isolated from untreated skin, chemically induced hyperplastic skin and chemically induced tumors from shaven haired mice (B). Reduction of *Lgr6* in chemically induced tumors compared to the untreated and hyperplastic skin was not significant (p=0.07). Error bars are SEMs.

As the wt *Lgr6* allele appeared not to be subjected to *de novo* promoter hypermethylation, it would be transcribed normally. Hence, we performed qPCR to investigate *Lgr6* mRNA expression in the skin tumors and check whether it was lost or reduced when compared to untreated or hyperplastic skin (the *EGFP-Ires-CreERT2* cassette was inserted in exon 1 of the *Lgr6* gene and blocked transcription of this gene [6]). In UV tumors from *Lgr6-EGFP-Ires-CreERT2/R26R-LacZ* and wt mice, we found a reduction of *Lgr6* expression when compared to untreated and hyperplastic skin (Figure 8A), in particular relative to *Ki67* expression (p= 0.004, see Supplementary Figure S3). Therefore, the qPCR analysis confirmed that Lgr6+ stem cells were lost from the pool of cycling cells (Ki67+) in UV-induced tumors. Although there was a trend towards lower expression of *Lgr6* in chemically induced tumors, the difference was not consistently significant (p=0.07 in haired, Figure 8B; p=0.04, in hairless mice, data not shown). In contrast to hairless mice, where *Ki67* expression was rather constant from untreated skin through UV- or TPA-treated skin up to and including tumors, shaven haired skin showed increased *Ki67* expression in UV-, TPA-treated skin and tumors (Supplementary Figure S4) *Lgr6* showed a relative decrease with increasing *Ki67*, again indicating a relative loss of Lgr6+ cells from the pool of cycling Ki67+ cells which apparently expanded in response to TPA (*Lgr6/Ki67* was significantly higher in untreated skin than in skin and tumors from TPA-treated haired mice, p=0.04, see Supplementary Figure S4).

## DISCUSSION

In this study we showed that Lgr6+ stem cells repopulate the IFE after a UV overexposure, and became much less frequent in the IFE after sub-acute chronic UV exposure, as did their progeny. Lgr6+ cells did not appear to become the tumor-initiating cells (TICs) in UV carcinogenesis, nor in two-stage chemical carcinogenesis.

To our knowledge we are the first to report that hairless mice with aberrant hair follicles do express *Lgr6*. As in haired mice, the Lgr6+ stem cells (EGFP+) are abundantly present in the interfollicular epidermis (IFE) and also present in the hair follicles (HFs) (Figure 1). In hairless mice most of the HFs are dysfunctional and the Lgr6+ cells reside at the bottom of the HF remnants in the region of the sebaceous glands. The fraction of EGFP+ cells (20 - 25%), which we established by area in epidermal sheets from hairless mice, was similar to the fraction of basal cells reported by Füllgrabe et al. for haired mice [9].

This base line abundance of EGFP+ cells in the IFE provided an excellent initial condition for lineage tracing by expression of LacZ after tamoxifen-induced activation of Cre. Both in shaven haired and hairless mice we observed a transient elimination of Lgr6+ stem cells (EGFP+) from the IFE, and a corresponding reduction in their progeny [1 – 3 days after UV overexposure]. As found previously in wounding [6, 9], the IFE was repopulated with Lgr6+ stem cells from the HF and, in contrast with unexposed controls, most of the progeny thereafter was associated with HFs. It was noted that the response in hairless mice appeared to evolve more rapidly than in haired mice, which corresponded to our earlier observations of a more rapid expulsion of apoptotic basal cells and stronger hyperplastic response in the hairless mice (see Supplement of ref [19]). This appears to parallel the observation under chronic UV exposure (see Supplement to [19]), that the epidermal hyperplasia was stronger in hairless than in haired mice.

Two months after the UV overexposure Lgr6 stem cell progeny was still observed in the IFE which confirmed that the Lgr6+ cells were indeed genuine stem cells (again similar to what was observed after wounding [6]). In shaven haired and hairless mice, daily sub-acute UV exposure led to a persistent decrease in Lgr6+ stem cells in the IFE (from >20 to <5%, Figure 5G) whereas the population in the HFs was not affected. The corresponding Lgr6 progeny was even more strongly reduced in the IFE and became exclusively associated with HFs. Evidently, under chronic UV exposure the scarce interfollicular Lgr6 progeny originated mainly from the HF, and in the IFE the Lgr6+ stem cells were most likely outcompeted by other interfollicular stem cells.

Liao and Nguyen [7] observed a juxtaposition of Lgr6+ stem cells and cutaneous nerve endings, and went on to demonstrate a dependency of the former on the latter. As UV has been reported to reduce epidermal nerve endings and dermal nerve fibers [20], this effect may underlie the depletion of Lgr6+ stem cells we observed in the chronically UV-exposed skin. Interestingly, Fullgrabe et al [9] found Lgr6+ stem cells from haired mice to express genes for axon guidance. These results would point at a reciprocal relationship between Lgr6+ stem cells and nerve endings, and thus, a UV-induced loss of Lgr6+ stem from the epidermis could lead to a loss of cutaneous nerve endings.

With lineage tracing from the start of the UV carcinogenic regimen, the Lgr6 progeny in the hyperplastic IFE between tumors appeared to be very similar to that observed earlier after 4 – 8 weeks of daily sub-acute UV exposure. However, the Lgr6 progeny in between chemically induced tumors appeared in large clusters contiguous with multiple HFs per cluster, suggesting that progeny from neighboring HFs had merged into large LacZ+ “islands”. Many HFs remained blank (LacZ-) and were surrounded by LacZ+ progeny in the IFE. This would suggest that progeny from interfollicular Lgr6+ stem cells may also have merged with these LacZ+ islands, outcompeting unlabeled progeny from blank HFs. Thus, the TPA tumor-promoting regimen appears to have had a totally different effect on Lgr6+ stem cells and their progeny in the IFE than the UV regimen.

The tumors themselves were devoid of Lgr6+ stem cells (no EGFP detected) and their progeny, except for some LacZ positivity in rare and small patches in terminally differentiated parts of the tumors – most likely inclusions of normal cells in the tumor mass. No tumor was raised from the lineage of the initially abundance of EGFP+ cells, i.e. none of the tumors was a clonal expansion of LacZ+ cells (which should have yielded a blue staining from cleaved X-gal throughout the tumor mass). The absence of Lgr6+ cells as driver cells in the tumor was further confirmed by starting lineage tracing after tumors had developed. Again, no progeny was detected in the tumors (progeny in surrounding skin constituted an internal positive control).

Because of these negative results in the tumors we investigated whether the reporter constructs in the mice were silenced, i.e whether the promoters of the *EGFP-CreERT2* cassette and *LacZ* were hypermethylated. The *Rosa* (*R26R)* promoter of *LacZ* was not methylated. Therefore, once the *LacZ* expression was switched on in Lgr6+ cells it was not affected by methylation and persisted in the progeny. If tumors arose in the lineage of initially abundantly present Lgr6+ stem cells this should have resulted in LacZ+ tumor masses. The *Lgr6* promoter, on the other hand, appeared stochastically methylated in the *Lgr6-EGFP-Ires-CreERT2* mice. As this methylation was never complete and it did not occur in wt mice we inferred that the methylation was directed at the *Lgr6* promoter of the *EGFP-CreERT2* cassette; it is known that insertion of foreign DNA can trigger *de novo* methylation [21]. This methylation could impair expression of the *EGFP-CreERT2* cassette and thus cause a loss of *EGFP* and *CreERT2* expression in Lgr6+ stem cells. As the methylation appeared to increase toward the end of the carcinogenic regimens (up to 100% of the samples with detectable methylation), both in tumors and uninvolved hyperplastic skin, detection of Lgr6+ stem cells and starting up lineage tracing could have been censored. Despite this possible censoring by methylation, starting up lineage tracing did yield positive results in the skin neighboring the tumors but not in the tumor bulk.

To check expression of *Lgr6* mRNA directly we performed qPCR. In hairless mice *Lgr6* expression was reduced in UV-induced tumors (Figure 8A) (also from wt mice) and in chemically induced tumors when compared to control and hyperplastic skin, especially relative to *Ki67* expression (Supplementary Figure S4). The latter would indicate a loss of Lgr6+ cells from the pool of proliferative (Ki67+) cells. The same tendency was observed for chemically induced tumors in shaven haired mice (Figure 8B). But the effect was obscured by an increase in the pool of cycling cells in TPA-treated skin and ensuing tumors, as reflected by an increase in *Ki67* expression. Relative to *Ki67* expression, *Lgr6* was significantly decreased when compared to control skin (Supplementary Figure S4). *Lgr6* expression provided no indication of an enrichment for Lgr6+ cells in the skin tumors raised by exogenous carcinogenic agents. In line with our data, Jensen e.a. [22] found overexpression of SRSF6 in SCCs, and this overexpression induced epidermal hyperplasia with a reduction in Lgr6+ cells.

Analyses of gene expression profiles of human SCCs have provided no indication of enrichment of Lgr6-expressing cells [23, 24]. This is in apparent agreement with our present results on experimentally (UV-) induced SCCs.

We conclude that the actively proliferating Lgr6+ stem cells are not targeted in UV or chemical carcinogenesis. Instead, quiescent stem cells are more likely to play an important role in skin carcinogenesis. Earlier it has been established in chemical carcinogenesis experiments that K15+/CD34+ quiescent stem cells in the bulge of the hair follicle are targets for transformation into TICs [25, 26]. We found that proliferating Lgr5+ stem cells in the hair follicle are not targeted in two-stage chemical skin carcinogenesis; like proliferating Lgr6+ stem cells in the present study, they do not appear to become TICs [19]. Moreover, we recently found that DNA damage-retaining quiescent stem cells in the IFE are linked to the initiation of persistent skin tumors by low level UV exposure [13]. Overall, these data suggest that the quiescent stem cells are more likely to become TICs by carcinogenic insults than continuously proliferating stem cells, such as Lgr6+ cells.

## MATERIALS AND METHODS

### Mice

*Lgr6-EGFP-Ires-CreERT2* (a kind gift of Prof. Hans Clevers) and Cre reporter *R26R-LacZ* mice (Jackson Laboratories, Bar Harbor, USA) were crossed to incorporate the LacZ reporter under the control of the Rosa (*R26R*) promoter for lineage tracing upon administering tamoxifen [6]. These *Lgr6-EGFP-Ires-CreERT2/R26R-LacZ* mice were also backcrossed into a hairless background using Crl:SKH1-HR hairless albino mice (Charles River, Sulzfeld, Germany).

Both male and female mice entered the experiments at 6-10 weeks of age (for each time point in the hyperplasia and ablation experiments n= 4). They were kept individually in Macrolon type 1 cages at 25 ± 2°C and about 50% humidity in a 12 hours light-12 hours dark cycle during experiments. The room in which the mice were kept and experiments were performed was illuminated by fluorescent tubes that did not emit any UV radiation. Standard chow and tap water were available ad libitum. As legally required, all mouse experiments were performed with the approval of the Leiden University Medical Centers's ethics committee (approval number DEC 10229).

### Experimental outline

A schematic overview of the experiments including time points of administering tamoxifen and of taking samples is presented in Supplementary Figure S1.

### Cre activation by Tamoxifen

Mice received three i.p. injections of tamoxifen (5 mg/injection, T5648 Sigma-Aldrich, Zwijndrecht, The Netherlands) over three days to activate lineage tracing (LacZ-producing cells) immediately prior to the start of the ablation and hyperplasia experiments. In the tumorigenesis experiments mice were divided into two groups: one group received tamoxifen injections at the start of the experiment, before the carcinogenic regimen, and the other group received tamoxifen injections when two tumors ≥4 mm developed. The mice in this last group were sacrificed 2-3 weeks after activation of lineage tracing.

### UV radiation

Philips TL-12/40W tubes were mounted over the cages and switched on and off automatically to deliver intended doses (output of 54% in UV-B, 280-315 nm, and 46% in UV-A, 315-400 nm). Under these lamps the minimal edema/erythemal dose (MED) for a naïve (previously unexposed) skin was determined to be 900 and 500 J/m^2^ UV for haired and hairless mice, respectively. To induce hyperplasia, mice were irradiated daily with 1 MED for 4-8 weeks, which induced no sign of sunburn (mice adapt to UV exposure). This same dose was used in the UV carcinogenesis experiments, where mice were daily irradiated until they had at least two ≥4 mm tumors. UV carcinogenesis was only performed with hairless mice (n= 13, 5 for early induction of lineage tracing and 8 for late induction) as shaven haired mice (C57BL6) in our laboratory started wounding themselves by severe scratching after months of chronically UV exposure, before developing any skin tumors [27].

For the overexposure experiments we used a higher dose that was just tolerable (no wounds) but largely ablated the basal layer of the epidermis (for haired mice 3.6 MED and for hairless mice 5 MED, 3.2 and 2.5 kJ/m^2^ UV respectively; i.e. quite similar).

### DMBA and TPA applications

For chemical carcinogenesis mice (haired: n=4 for early induction of lineage tracing and n=6 for late induction; hairless n=5 for early induction and n=4 for late induction) received a DMBA application (100 μg, 7,12-Dimethylbenz[a]anthracene, D3254, Sigma-Aldrich), on day 1 and from day 8 onward TPA treatment (12-O-tetradecanoylphorbol-13-acetate, P8139, Sigma-Aldrich) twice a week until at least two ≥4 mm tumors developed. With each application, 10μg TPA in 100μl acetone was applied on approximately 6 cm^2^ of dorsal skin using a fine brush. Haired mice were priorly shaven to remove hair covering the dorsal skin.

### Tissue preparation

Mice were sacrificed by CO_2_ asphyxiation. Dorsal and ventral skin was excised and segmented for use in different histochemical stainings. Samples for the anti-Caspase-3 and β-galactosidase staining were embedded in Tissue-tek, snap frozen in liquid nitrogen and stored at -80°C until sectioning and staining. Samples for the anti-EGFP staining were fixed overnight in PBS-buffered 4% formaldehyde solution (ROL1642810, Addedpharma, Oss, The Netherlands) and embedded in paraffin. Whole mount skin biopsies for LacZ lineage tracing were cut into pieces of 5x5 mm and incubated in 20 mM EDTA (Baker, Deventer, The Netherlands) in PBS O/N at 37°C. The next day, they were washed with PBS, fixed in PBS-buffered 4% formaldehyde solution for 5 min and incubated O/N with X-gal solution (1 mg/ml X-gal, 5 mM ferrothiocyanide, 5 mM ferrithiocyanide, 2 mM MgCl_2_ in PBS). After incubation they were embedded in Kaisers glycerin. Epidermal sheets for LacZ staining were treated similar to the whole mounts. Skin was excised, fatty tissue was removed, and samples were submerged for 2 hrs in 20 mM EDTA in PBS at 37°C. After incubation the epidermal sheet was pealed from the dermis and was fixed for 5 min in PBS-buffered 4% formaldehyde solution and stained according to the protocol of the whole mount biopsies. Clusters of LacZ positive cells in an epidermal sheet were counted in 10 microscopic frames at 10X objective (2 mm cross section).

Tumors were either snap frozen in liquid nitrogen or fixed in PBS-buffered 4% formaldehyde solution and embedded in paraffin.

### Immunohistochemistry

#### EGFP staining

Paraffin samples were cut at 5μm thickness and incubated at 60°C O/N. The next day, they were dehydrated and antigen retrieval was performed with antigen unmasking solution (H-3300, Vector Laboratories, Inc Burlingame, USA) in a pressure cooker for 5 min. Non-specific binding was blocked with PBS/0.1% Tween/1% BSA for 2 h followed by incubation with anti-EGFP (1:200, ab139070, Abcam, Cambridge, UK) at 4°C O/N. The sections were incubated with secondary antibody Alexa Goat anti-chicken 488 (1: 250, Life technologies, Bleiswijk, The Netherlands) and nuclei were stained with Dapi for 5 min (1:3000, D1306, Invitrogen, Bleiswijk, The Netherlands). The sections were mounted with Vectashield mounting medium for fluorescence (H-1000, Vector Laboratories, Inc. Burlingame, USA).

#### β-galactosidase/LacZ staining

Cryosections were cut at 6 μm thickness and fixed with 4% PFA in PBS (P6418, Sigma Aldrich, Steinheim, Germany) for 10 min at RT. Sections were washed with PBS and with Rinse solution (2 mM MgCl_2_, 0,01% NP40 in PBS) and incubated O/N at 37°C with β-galactosidase staining solution (5 mM K_3_Fe(CN)_6_, 5 mM K_4_Fe(CN)_6_·3H_2_O, 1 mg/ml X-gal in Rinse solution). Sections were washed with rinse solution and counterstained with haematoxylin and eosin. Sections were dehydrated and embedded in Depex (18243.01, Serva Electrophoresis GmbH, Heidelberg, Germany).

Images were acquired using a Zeiss Axioplan 2 microscope with 10x and 20x objectives, Axiocam camera and dedicated software for immunohistochemistry. For fluorescent pictures a Leica DM 5000B Microscope was used with 5x, 10x and 20x objectives and a Leica DFC300 FX Camera with dedicated software. Final pictures were formatted in Adobe Photoshop CS6 or Adobe Illustrator CS6 and representative cases are presented in Results.

### CLSM

For confocal microscopy of fluorescent EGFP+ cells epidermal sheets were prepared by incubating skin for 2 hrs in 20mM EDTA (Baker, Deventer, The Netherlands) in PBS at 37°C. After incubation the epidermis and dermis were separated. The epidermis was fixed for 10 min in 4% paraformaldehyde and embedded in Vectashield containing DAPI (H1200, Vector Laboratories, Burlingame, U.S.A). Pictures were taken using the LSM 700 confocal microscope (Zeiss) with the LCI Plan-Neofluar 25x objective/0.8 Imm Korr DIC M27.

The area of EGFP+ cells was calculated using the CLSM data and ImageJ. We measured the area (number of pixels) of DAPI+ cells (after smoothening the nuclear fluorescence to create a contiguous area of DAPI+ cells) in a z-stack plane and the area of EGFP+ cells in the same plane for multiple samples/frames from 2-3 mice. From those data we calculated the percentage of the total area that was EGFP+.

### Methylation specific melting curve analysis (MS-MCA)

DNA was isolated from frozen tissue using the DNeasy kit (Qiagen, Hilden, Germany) according to manufacturer's protocol. Bisulphite conversion was performed on 1 μg DNA using the EZ DNA methylation kit (Zymo Research, Orange, CA). Bisulphite primer sequences were designed to amplify part of the CpG island located in the gene body of *Lgr6*, +245 to +371 bp downstream of the TSS (NM_001033409.3). Primers were developed in such a way that both methylated as well as unmethylated sequences were amplified using the same bisulphite-treated DNA as PCR template (Supplementary Table SI). Amplification was performed with iQ SYBR Green Supermix on a CFX384 Touch Real-Time PCR Detection System (Bio-Rad, Veenendaal, The Netherlands) using a touchdown PCR protocol with the following parameters: denaturing at 95°C for 30 seconds (ramp rate at 2°C per second), followed by 7 cycles of annealing at 65°C to 58°C for 40 seconds (with a 1°C decrement per cycle) and extension at 72°C for 40 seconds. For the following 33 cycles annealing was performed at 60°C. The last extension step at 72°C was extended to 3 minutes. Following amplification, melting curves were acquired during a linear temperature transition from 65 to 90°C with increments of 0.2°C per 10 seconds. Bisulfite primer sets were validated for gene specificity on bisulfite treated methylated CpGenome Universal Methylated Mouse DNA Standard (Millipore, Amsterdam, The Netherlands) and unmethylated wt C57BL/6 mice DNA as reference control sample. The presence of methylated DNA was detected through melting peaks with higher melting curve temperatures compared to unmethylated DNA as obtained from the reference control samples. Melting peaks were detected by CFX manager 3.1 software (Bio-Rad) and average melting temperatures were calculated for these recognized peaks. A promoter was considered methylated if melting temperatures were exceeding half of the maximum temperature difference as determined for the reference control samples.

### Quantitative real-time PCR (qPCR)

Total RNA was isolated from frozen skin and tumors using the RNeasy mini kit (74106, Qiagen, Hilden, Germany) including on-column DNase treatment. cDNA was synthesized from 1 μg total RNA using the iScript™ cDNA Synthesis Kit (170-8891, Biorad, Veenendaal, The Netherlands) according to manufacturer's instructions. qPCR for Lgr6 mRNA was performed using the SYBR Green Supermix (Bio-Rad) on the CFX384™ real-time PCR detection system (Biorad). Cycle parameters were as follows: Hot start for 3 minutes at 95°C; denaturation for 15 seconds at 95°C, annealing and extension for 30 seconds at 60°C for 40 cycles. Specificity of the PCR products was confirmed by melting curve analysis. Data was normalized against reference genes Cyc1 and Ddx52 (see Supplementary Table SIII for primers used) using the ΔΔCq method [28] and is presented as relative mRNA expression.

### Data processing and statistical analyses

Statistical significant differences in EGFP+ areas and in mRNA expression were determined by the Mann-Whitney test (in Graphpad Prism 6). Statistical significance was set at p≤0.05. Means were given with standard error of the mean (SEM) unless otherwise stated. Graphs were generated in Graphpad Prism 6 and formatted in Adobe Illustrator CS6.

## SUPPLEMENTARY FIGURES AND TABLES


