# Supplementary material for: Lgr6+ stem cells and their progeny in mouse epidermis under regimens of exogenous skin carcinogenesis, and their absence in ensuing skin tumors
Source: Oncotarget. 2016 Nov 17;7(52):86740–54. doi: 10.18632/oncotarget.13436 (PMC5349950; doi:10.18632/oncotarget.13436)
Supplement: Supplementary file 1 [file oncotarget-07-86740-s001.pdf]

Lgr6+ stem cells and their progeny in mouse epidermis under regimens of exogenous skin carcinogenesis, and their absence in ensuing skin tumors

SUPPLEMENTARY INFORMATION

METHODS

Active Caspase-3 Staining

Cryosections were cut at 6 µm thickness and fixed in acetone containing 0.3% H_2O_2. The sections were blocked with 2% Normal Human Serum (NHS) for 20 minutes. After blocking, the sections were incubated overnight at 4°C with anti-active caspase-3 (1:100, ab 2302, 3509322, Abcam, Cambridge, UK). The next day the sections were incubated for one hour with Goat-anti-Rabbit (IgG)-biotin (1:300, Vector Laboratories, Inc Burlingame, USA) followed by streptavidin (1:100, RPN1051v, GE Healthcare UK Limited) for 45 minutes. The staining was visualized with 20 mg 3,3'-Diaminobenzidine (D5905, Sigma-Aldrich, Zwijndrecht, The Netherlands) in 100ml of PBS and 100 µl of H_2O_2. Sections were counterstained with haematoxylin and mounted in Kaisers' glycerin.

SUPPLEMENTARY FIGURES AND TABLES

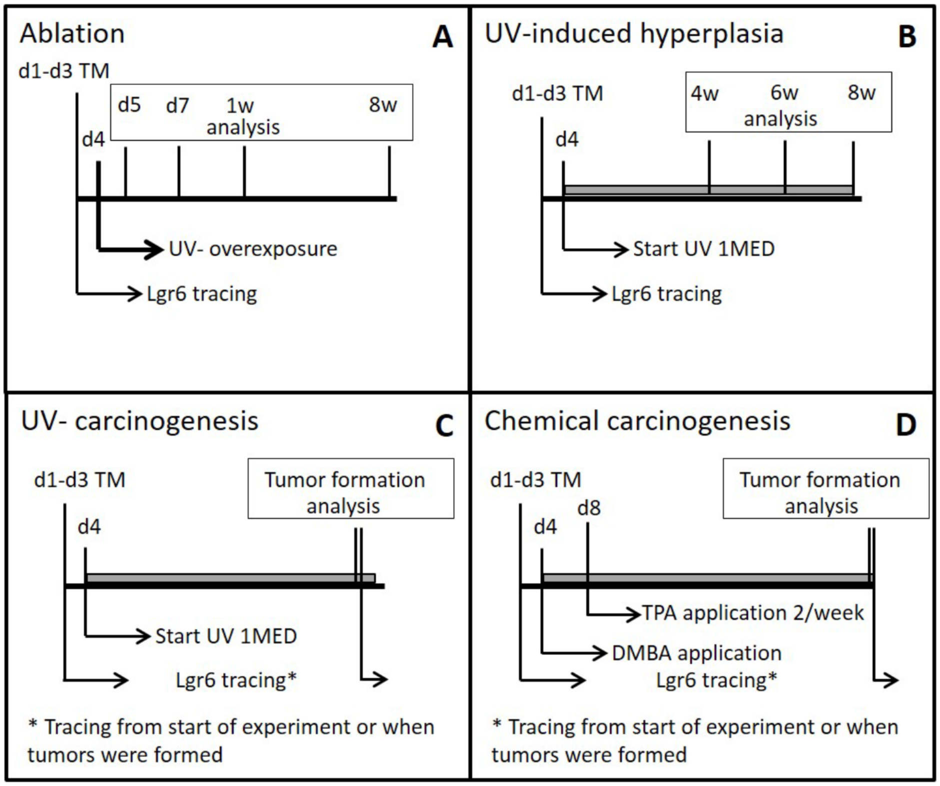

Supplementary Figure S1: Experimental outline. Time lines of the experiments including time points of sample collection for analyses. TM= tamoxifen injection, d= day, w=week. In experiments **A** and **B.** the lineage tracing started at the beginning of the experiment. In experiment **C** and **D.** the tracing started either at the start of the experiment or when tumors were formed.

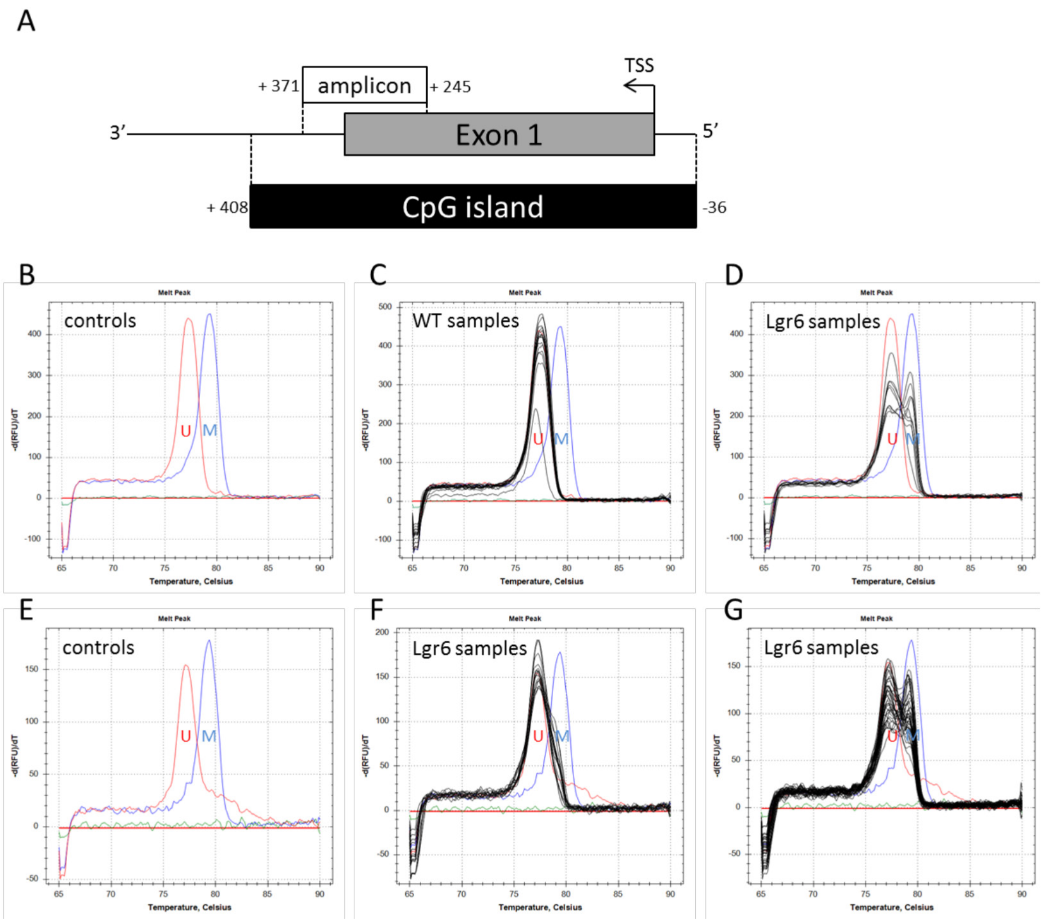

Supplementary Figure S2: Methylation-specific melting curve analysis (MS-MCA). A Schematic depiction of the CpG island in relation to the promoter and gene body and amplicon for MS-MCA relative to the transcription start site (TSS) of *Lgr6* **A.** MS-MCA on methylation control samples resulting in a melting temperature of 77.4 °C for unmethylated (U) and 79.4 °C for methylated (M) bisulphite converted DNA (B+E). In the first experiment **B-D.** samples from *Lgr6* transgenic mice (D) and mice with only wildtype (wt) *Lgr6* loci were tested (C). The wt samples were all unmethylated, however several *Lgr6* samples showed aberrant promoter methylation, demonstrated by the presence of both a methylated as well as an unmethylated fraction. In a second experiment only *Lgr6* samples were tested **E-G.**, 15 out of 54 were unmethylated (F) and 39 out of 54 showed aberrant methylation (G). See also supplementary table S2 for the tested samples.

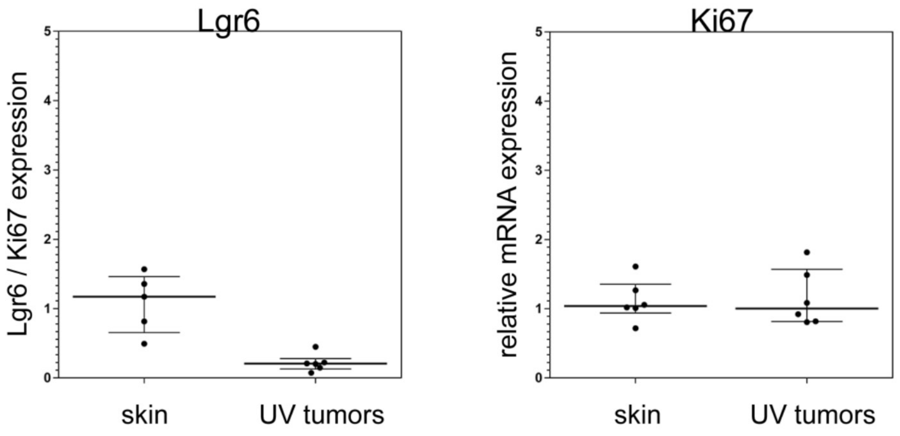

Supplementary Figure S3: Normalized *Lgr6* and *Ki67* expressions in wildtype SKH mice. Left panel: ratio of *Lgr6* expression over *Ki67* expression. Right panel: normalized *Ki67* expression (proliferating cells). Relative *Ki67* expression was equal in tumors induced by UV and in skin (untreated and hyperplastic skin) samples. However, *Lgr6* expression was significantly reduced in tumors (p=0.004, Mann-Whitney test). Lines depict median with interquartile range.

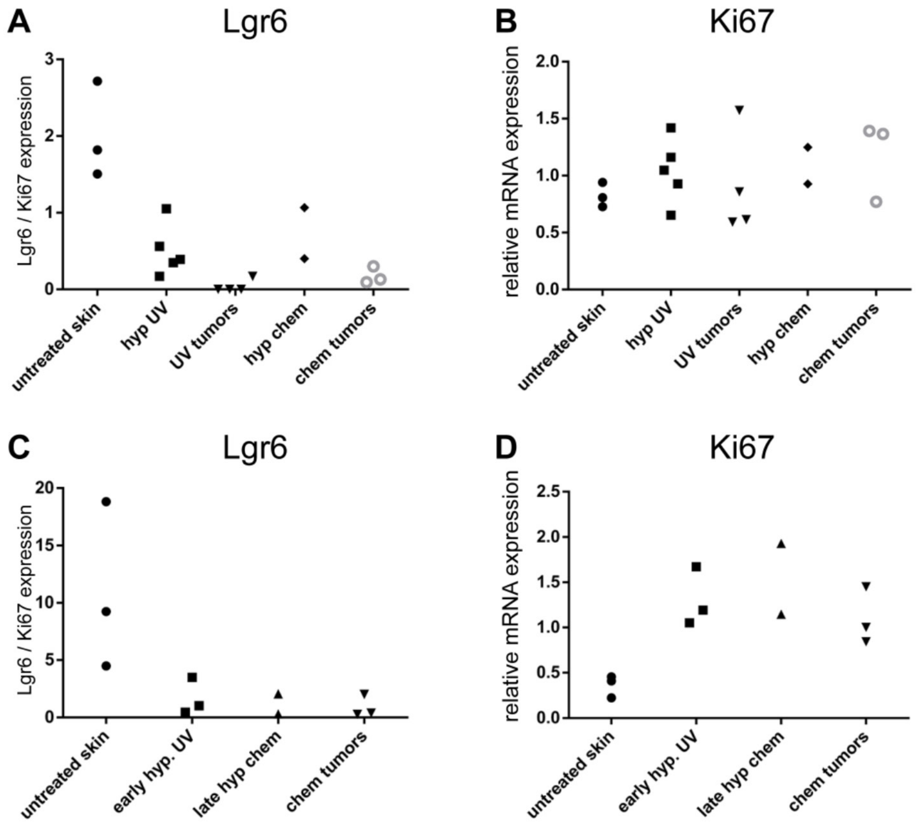

Supplementary Figure S4: Normalized *Lgr6* and *Ki67* expressions in hairless A+B. and haired C+D. *Lgr6* mice. The expression of *Lgr6* in each sample was divided by the corresponding expression of *Ki67* (A+C). Tumors and skin samples showed a similar expression of *Ki67* in hairless mice (B), while in haired mice the *Ki67* expression in untreated skin was reduced compared to the other samples (D). In hairless mice the *Lgr6* expression relative to the *Ki67* expression in UV tumors was lower than in untreated skin, though not statistically significant (p=0.057, Mann-Whitney test). In chemically induced tumors and hyperplasia samples compared to untreated skin this expression was significantly reduced (p=0.036). In haired mice the ratio of *Lgr6* over *Ki67* expression in untreated skin was higher than in chemically induced late hyperplasia and chemo tumors (p=0.036).

Supplementary Table SI: Primers for methylation assay

Gene	Primer sequence (5'-> 3')	Amplicon size	Number of CpG's	Relative to TSS	Tm unmethylated amplicon	Tm methylated amplicon
Lgr6	GGTATTAGTAGGTGA GTATTTATAGGTAAG	127 bp	6	-36 to +408	77.4°C	79.4°C
	ACTCCCTACCAC TACCAAAAAAAC					
Rosa	GGTGTAATGGTGT GTAAAGGTAGTTG	192 bp	8	-684 to -493	78.8°C	80.6°C
	CCTAAATCAAAATA AAAATCCAACAAA					

Supplementary Table SII: Samples tested for promoter hypermethylation

sample group	# tested (n)	# Aberrantly methylated Lgr6 promoter (n)	% samples aberrantly methylated
Skin untreated (C57BL/6 mice)	5	0	0
skin untreated (SKH-1 mice)	2	0	0
skin untreated (haired *Lgr5-EGFP-Ires-CreERT2/R26R-LacZ* mice)	3	0	0
skin untreated (haired *Lgr6- EGFP-Ires-CreERT2/R26R-LacZ* mice)	8	3	38
skin untreated (hairless *Lgr6- EGFP-Ires-CreERT2/R26R-LacZ* mice)	8	4	50
skin treated with UV 30 days 1 MED (SKH-1 mice)	4	0	0
skin treated with UV 4-8 weeks 1 MED (*haired Lgr6- EGFP-Ires-CreERT2/R26R-LacZ mice*)*	8	2	25
skin treated with UV 4-8 weeks 1 MED (*hairless Lgr6- EGFP-Ires-CreERT2/R26R-LacZ mice*)*	6	4	67
skin treated with TPA 6 weeks (haired *Lgr5-EGFP-Ires-CreERT2/R26R-LacZ mice*)	3	0	0
inter-tumoral skin UV-carcinogenesis (hairless *Lgr6- EGFP-Ires-CreERT2/R26R-LacZ mice*)	4	3	75
inter-tumoral skin chemical carcinogenesis (haired *Lgr6- EGFP-Ires-CreERT2/R26R-LacZ mice*)	6	6	100
inter-tumoral skin chemical carcinogenesis (hairless *Lgr6- EGFP-Ires-CreERT2/R26R-LacZ mice*)	6	5	83
Tumors: UV-carcinogenesis (SKH-1 mice)	6	0	0
Tumors: UV-carcinogenesis (hairless *Lgr6-EGFP-Ires-CreERT2/R26R-LacZ mice*)	9	8	89
Tumors: chemical carcinogenesis (haired *Lgr5-EGFP-Ires-CreERT2/R26R-LacZ mice*)	6	0	0
Tumors: chemical carcinogenesis (haired *Lgr6-EGFP-Ires-CreERT2/R26R-LacZ mice*)	9	9	100
Tumors: chemical carcinogenesis (hairless *Lgr6- EGFP-Ires-CreERT2/R26R-LacZ mice*)	9	9	100

*no significant difference between skin treated with UV of haired and hairless mice (p=0.28; fisher exact test)

Supplementary Table SIII: Primers used for qPCR

Gene	Primer sequence (5'-> 3')	Amplicon size	Exonic Location	Accession No.	Amplification efficiency	Correlation (R^2)
Cyc1	GATGTCGCCA CCTTCCTTCGC	136	6, 7	NM_025567.2	98.6%	0.99
	GGACTGACCAC TTATGCCGCTTC					
Ddx52	CACAGCAGCAG CGAGATAACAC	177	10-11, 11-12	NM_030096	107.0%	0.99
	CTCTTCCAGTCCG ACCTATCCTATG					
Lgr6	TCAGGGAACC ACCTCTCACA	113	3, 4	NM_001033409	92.0%	0.95
	CATAGTGCCT CTGCTGGGAT					
Mki67	CAGAAGAAATG GAAGCCAAAA	110	12, 13	NM_001081117.2	96.5%	0.99
	TGCAGATGCA TCAAACTTGG					
